# A semitendinosus with adjustable button graft construct in patients undergoing hamstring ACL reconstruction results in improved knee flexor strength symmetry and less donor site pain and morbidity: Outcomes from the DOSTAR randomized controlled trial

**DOI:** 10.1002/ksa.12698

**Published:** 2025-05-12

**Authors:** Adam M. Lawless, Jay R. Ebert, Peter K. Edwards, Shahbaz S. Malik, Peter S. E. Davies, Peter A. D'Alessandro

**Affiliations:** ^1^ Fiona Stanley and Fremantle Hospitals Group South Metropolitan Health Service Perth Western Australia Australia; ^2^ School of Human Sciences (Exercise and Sport Science) University of Western Australia Perth Western Australia Australia; ^3^ HFRC Perth Western Australia Australia; ^4^ Orthopaedic Research Foundation of Western Australia Perth Western Australia Australia; ^5^ School of Allied Health Curtin University Perth Western Australia Australia; ^6^ Worcester Acute Hospitals NHS Trust Worcester UK; ^7^ School of Surgery University of Western Australia Perth Western Australia Australia

**Keywords:** adjustable button, anterior cruciate ligament reconstruction, donor site morbidity, functional outcomes, gracilis, hamstring autograft, semitendinosus

## Abstract

**Purpose:**

To compare donor site morbidity and patient‐reported outcome measures (PROMs), knee laxity and early strength and functional outcomes, following anterior cruciate ligament reconstruction (ACLR) using a semitendinosus (ST) tendon autograft with suspensory adjustable button tibial fixation or semitendinosus–gracilis (STG) autograft with tibial screw fixation.

**Methods:**

While 153 patients were prospectively recruited and randomized to an ST or STG autograft, 131 (62 ST and 69 STG) were retained following ACLR and followed post‐surgery. Standardized surgical techniques were employed, varying only in graft selection and fixation. The primary study outcomes were early hamstring pain, donor site morbidity and strength recovery. However, several outcomes were assessed pre‐surgery and at 3 and 6 months, encompassing the Donor‐site‐related Functional Problems following Anterior Cruciate Ligament Reconstruction (DFPACLR) score, Visual Analogue Scale (VAS) for hamstring pain and other commonly employed PROMs, anteroposterior laxity (KT‐1000), isokinetic hamstring and quadriceps strength, hop testing, complications and re‐operations. Intention‐to‐treat analysis was performed using linear mixed models for continuous data and Mann–Whitney *U* tests where appropriate.

**Results:**

At 6 months, ST patients reported significantly lower hamstring pain (*p* < 0.001) and DFPACLR (*p* < 0.001) scores. A significantly higher (*p* < 0.001) peak knee flexor strength limb symmetry index (LSI) was observed for the ST group, though no other group differences in side‐to‐side laxity, hop tests or other normalized strength measures or LSIs were observed.

**Conclusions:**

ACLR using an ST autograft resulted in less donor site pain and morbidity, and improved knee flexor strength symmetry at 6 months, while demonstrating comparable functional outcomes to the STG autograft.

**Level of Evidence:**

Level 1 prospective, double‐blinded, randomized controlled trial.

**Abbreviations**
ACLR: anterior cruciate ligament reconstruction
BMI: body mass index
CKRS: Cincinnati Knee Rating System
DFPACLR: Donor‐site‐related Functional Problems following Anterior Cruciate Ligament Reconstruction
IKDC: International Knee Documentation Committee
LET: lateral extra‐articular tenodesis
LKS: Lysholm Knee Score
LSI: limb symmetry index
MRI: magnetic resonance imaging
PROMs: patient‐reported outcome measures
RCT: randomized controlled trial
ROM: range of motion
SHD: single horizontal hop for distance
SMD: standardized mean difference
ST: semitendinosus
STG: semitendinosus–gracilis
VAS: visual analogue scale
VAS‐F: visual analogue score for pain frequency
VAS‐S: visual analogue score for pain severity

## INTRODUCTION

Rupture of the anterior cruciate ligament (ACL) is a common knee injury [17], often necessitating ACL reconstruction (ACLR) to restore stability and function, facilitating a successful return to sports activities [3, 21]. Hamstring autograft remains a popular graft choice in those undergoing ACLR [1, 2] and, in Australia, is the chosen method for primary ACLR in over 90% of knee reconstructions [9]. This preference stems primarily from the perceived reduced donor site morbidity, including decreased incidence of anterior knee pain associated with bone‐patella‐tendon‐bone and quadriceps tendon autograft harvesting [24]. However, hamstring harvesting is not without its drawbacks. Significant donor site morbidity, including pain, recurrent hamstring strains and diminished knee flexion strength, has been documented [7, 11, 13, 24].

While advancements in surgical instrumentation and harvesting techniques have aimed to mitigate donor site issues [7], a key debate revolves around the necessity of harvesting both the semitendinosus (ST) and gracilis (G) tendons. Intuitively, harvesting a single tendon (ST) should reduce morbidity compared to harvesting both tendons (STG) due to less soft tissue trauma [1, 6]. Newer suspensory fixation techniques reliably allow for sufficient graft diameter using only the ST tendon [19], leading to the proposition that ST harvest is sufficient. Proponents suggest the spared gracilis tendon undergoes compensatory hypertrophy, providing structural and functional compensation, especially during deep flexion [4, 12]. This has been further supported by the suggestion that gracilis acts as a restraint to anterior tibial translation, enhancing the stability of the reconstructed ACL [25]. In addition, some evidence suggests that gracilis tendon harvest leads to greater knee flexion deficits, particularly at deeper knee angles [5, 12, 18]. However, much of the existing literature comprises underpowered and retrospective studies [1, 6, 12, 16], necessitating a large‐scale, prospective, randomized controlled trial (RCT) to appropriately investigate these harvesting techniques.

The primary study aim was to evaluate early hamstring‐related pain, donor site morbidity and isokinetic hamstring strength recovery, in community‐level patients undergoing ACLR via a single ST tendon autograft with suspensory button tibial fixation, versus a STG autograft with screw fixation. Secondary aims were to assess group differences in commonly employed patient‐reported outcome measures (PROMs), knee laxity, isokinetic quadriceps strength and functional outcomes. It hypothesized that within the first 6 months post‐surgery, ST ACLR with suspensory button tibial fixation versus STG ACLR with tibial screw fixation, would: (1) be associated with a significantly lower level of hamstring pain and donor site morbidity, (2) be associated with lower hamstring strength deficits and (3) be largely similar in other patient‐reported and objective measures including anteroposterior laxity, quadriceps strength and hop capacity.

## METHODS

This double‐blinded, prospective RCT was prospectively registered with the Australia New Zealand Clinical Trials Registry and approved by the relevant Human Research Ethics Committee. The primary aim was to evaluate early donor site morbidity and isokinetic hamstring strength recovery, though for completeness also assessed PROMs, side‐to‐side anteroposterior knee laxity, peak isokinetic quadriceps strength, and functional hop capacity, within the first 6 months in patients undergoing ACLR with an ST versus STG hamstring tendon harvest.

All patients who were scheduled to undergo ACLR via a single surgeon with fellowship training in sports surgery (PD), fulfilling the study inclusion criteria, were approached and subsequently recruited for the study. Patients were randomized to either an ST or STG autograft construct using a ‘random number sequence generator’, with the randomization sequence maintained by an independent researcher. Patients were included if they were 18–50 years of age and qualified for ACLR based on clinical examination and magnetic resonance imaging (MRI). Patients requiring additional procedures such as meniscal repair were included, as were those deemed candidates for lateral extra‐articular tenodesis (LET). Criteria for LET broadly aligned with the STABILITY trial [10], with those undergoing LET generally presenting with at least three of the following criteria: female, <25 years of age, seeking a return to pivoting sports, generalized ligamentous laxity, >5° hyperextension and a high‐grade pivot shift on clinical examination or examination under anaesthesia.

Exclusion criteria included unwillingness or unable to provide written consent specific to this study, body mass index (BMI) ≥ 40, revision ACLR or multi‐ligamentous reconstruction, and patients presenting with significant articular cartilage pathology that likely required concomitant surgical intervention. Patients who had undergone prior contralateral ACLR were included in the current study, provided they had been undertaken ≥5 years prior. Patients with established osteoarthritic changes (those with Outerbridge Grade 3 changes or above) were not candidates for study inclusion. For recruited patients, those who failed to meet a minimum required graft diameter of 8mm at the time of surgery, therefore necessitating the use of an alternative graft technique, were further excluded to ensure the patient still received a graft of a minimum 8mm diameter. This decision was made in accordance with the surgeon's preference, which is supported by findings from the Multicenter Orthopaedic Outcomes Network group and corroborated by data from the Swedish Ligament Registry [15, 23].

### Surgical technique and rehabilitation

All patients received a standardised anaesthetic regime including general anaesthesia in conjunction with an adductor canal block (20 mL of 0.2% ropivacaine), local anaesthetic infiltration of the hamstrings site (50 mL of 0.2% ropivacaine), with remaining local infiltration of the knee. The surgical technique was standardised across both groups, with variations only in graft selection. Donor hamstrings were harvested from the ipsilateral knee through an oblique anteromedial incision over the pes anserinus. Graft preparation was performed according to the patients' assigned study arm: patients received either a four‐strand quadrupled ST graft with an adjustable loop Ultra‐Button (Smith & Nephew) or a doubled STG graft with Bio RCI (Smith & Nephew) screw fixation for tibial fixation. While the graft constructs (ST and STG) were different, the methods of tibial fixation differed as outlined above. In brief, adjustable button fixation on the tibia allows for a short graft (quadrupled) harvest and only one tendon. The harvest of one tendon is not permitted with a screw as the longer graft required would result in inadequate graft diameter. All grafts employed a fixed loop Endobutton (Smith & Nephew) for femoral fixation. Arthroscopic evaluation was performed through the standard anteromedial and anterolateral portals. The femoral notch was carefully prepared, and independent femoral reaming was undertaken via the anteromedial portal while the knee was maintained in high flexion. Tibial preparation involved the use of a 55° angled drill guide, and both femoral and tibial tunnels were reamed to match the dimensions of the graft, which was soaked in vancomycin. The graft was then shuttled through the tibial tunnel into the knee and passed through the femoral tunnel under arthroscopic visualization until successful docking occurred. The graft was cycled through the range of motion (ROM) to ensure appropriate tensioning. Final arthroscopic assessments, including images with the knee in full extension, were obtained to confirm the absence of graft impingement or cyclops lesion. Meniscal repair and/or LET were performed as indicated based on arthroscopic findings, patient factors and pre‐operative assessment, as outlined earlier.

Rehabilitation over the first 6 weeks was standardised for both groups. Initially, this was focused on pain/swelling control, safe and appropriate ambulation, and exercises to improve knee ROM and quadriceps activation. Patients undergoing meniscal repair were placed in a hinge knee brace and restricted to partial weight bearing with crutches for 4–6 weeks. This was generally 20%–30% body weight for the first 2 weeks, progressing towards 50%–75% for a further 2 weeks, with subsequent progression toward full weight bearing from 4 weeks post‐surgery though also depending on the size, geometry and location of the tear. Patients without meniscal repair were not braced and permitted to weight bear as tolerated. Rehabilitation was not strictly standardized from 6 weeks post‐surgery and was undertaken under the direction of the patient's own therapist as this was a study designed to reflect community practice. Furthermore, for future RTS, patients were advised on key objective criteria that should be met before resuming sports, such as the restoration of full active knee ROM, as well as physical performance scores (quadriceps and hamstring strength, as well as single‐limb hop capacity) on the operated limb ≥90% of the contralateral limb. However, RTS prior to 6 months was not advocated or permitted, and no patient in the current study had RTS prior to the 6‐month assessment. Nonetheless, this was not the focus of the current study.

### Clinical outcome measures

Firstly, baseline patient characteristics were collected, including patient demographics (age, sex, body weight and BMI) and injury history (time from injury to surgery, whether the injured side was the dominant kicking limb, injury mechanism). A number of PROMs were undertaken pre‐surgery and at 3 and 6 months post‐surgery, including the International Knee Documentation Committee (IKDC) Subjective Knee Evaluation Form, the Lysholm Knee Score (LKS), the Cincinnati Knee Rating System (CKRS), and a Visual Analogue Pain Scale for knee pain frequency (VAS‐F) and severity (VAS‐S). Post‐operatively, a VAS for hamstring pain was collected, while the Donor‐site‐Related Functional Problems following Anterior Cruciate Ligament Reconstruction (DFPACLR) and Anterior Cruciate Ligament Return to Sport after Injury (ACL‐RSI) scores were administered specifically at 6 months. A patient satisfaction questionnaire was also employed at 6 months post‐surgery to assess each patient's level of satisfaction with the surgery overall, as well as their satisfaction with the surgery in relieving knee pain, improving the ability to perform normal daily activities and their ability to participate in recreational activities.

All patients underwent a formal knee laxity examination performed in the clinic by the senior author at 8 weeks and 4 months post‐surgery, specifically to assess rotatory laxity grading via pivot shift evaluation. Further to this, the KT‐1000 knee arthrometer (MEDmetric Corp.) was employed at 6 months post‐surgery to assess anterior tibial translation (mm) during a maximal manual test on both limbs. The single horizontal hop for distance (SHD, m) was also assessed at 6 months post‐surgery, as was peak isokinetic knee extensor and flexor strength (Nm). Isokinetic strength measures were presented normalized to body weight (Nm/Kg) as well as a limb symmetry index (LSI) compared with the non‐operated side. The SHD was presented normalized to height (m/m) and as an LSI. Finally, complications, adverse events and re‐operations were documented over the first 6 months.

### Statistical analysis

For this prospective RCT, a priori sample size power calculation was determined employing G‐Power. The primary outcome variable was post‐operative graft harvest site (hamstrings) pain using a VAS at 6 months post‐surgery. Based on an anticipated moderate effect of size (*d* = 0.50) in hamstring pain employing outcomes from a previous study investigating different hamstring graft harvest methods after ACLR, an estimated sample of 128 (64 per group) is required to reveal differences at alpha 0.05 with 80% power [7].

The primary method for data analysis was via intention to treat. Continuous data with baseline measures were analyzed using linear mixed models, to analyze between‐ and within‐group treatment effects at each follow‐up time point (3 and 6 months post‐surgery). The models were adjusted for baseline values of each outcome variable to ensure more accurate estimates, as well as age, sex, and concomitant meniscal repair and LET. Missing continuous data were accounted for by restricted maximum likelihood estimation within the linear mixed models. Linear mixed models, being a likelihood‐based estimation procedure, result in non‐biased estimates when data are missing at random. This approach estimates the likely values for the missing data based on the information contained in the observed data. Group, time (as a categorical variable), group by time, and baseline values of each outcome variable were included as fixed effects. Participants were included as a random effect to account for within‐person correlation. Effect sizes for all outcomes in the primary analysis were also calculated as standardized mean difference (SMD) from estimated marginal mean and standard error estimates and interpreted according to Cohen's criteria (small ≤ 0.2; moderate = 0.5; large ≥ 0.8).

Between‐group analyses for 6‐month outcomes, including hamstring pain, donor site morbidity, knee laxity, normalized peak knee extensor and flexor torque, limb symmetry indices (LSI), and single‐leg hop for distance, were performed using linear mixed models. Again, each model included participant ID as a random intercept to account for individual variability. Fixed effects included group (ST and STG), and covariates including age, sex, and concomitant meniscal repair and LET. Mann–Whitney *U* tests were used to determine a difference in satisfaction scores between groups at 6 months. Analysis was performed with SPSS (version 30). All tests were two‐tailed with alpha set at 0.05.

## RESULTS

**Figure 1:**
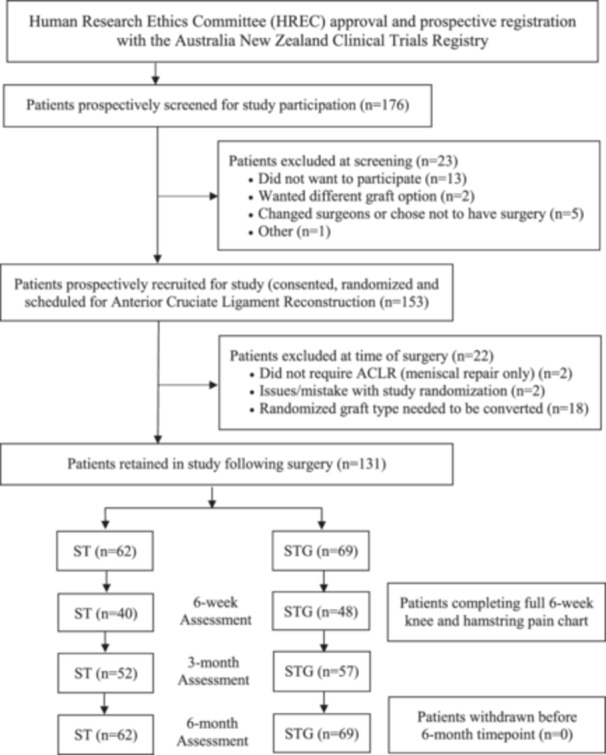
Flowchart demonstrating ethics approval and clinical trial registration, patient screening, recruitment, evaluation and loss to follow‐up over the study period, in patients randomized to the semitendinosus (ST) and semitendinosus/gracilis (STG) autograft groups.

A total of 176 patients were initially screened for participation in the study, of which 153 were successfully recruited and randomized (Figure 1). At the time of surgery, a further 22 patients were excluded, with the most common reason being an inadequate graft diameter <8 mm (*n* = 18) that required conversion (Figure 1). These patients required alternative tendon preparation techniques or the addition of the gracilis tendon, depending on their group allocation, to create a graft >8 mm. The method of augmentation was meticulously recorded. The remaining 131 patients that were retained in the study following surgery included 62 ST and 69 STG, of which 100% of patients were available for review at 6 months, with no loss to follow‐up (Figure 1). Patient, injury and surgery variables for the two groups are shown in Table 1.

**Table 1:** Baseline characteristics of the study population that were recruited, randomized to the semitendinosus (ST) and semitendinosus/gracilis (STG) autograft groups, underwent surgery and were retained in the study following surgery.

Variable	Measure	ST (*n* = 62)	STG (*n* = 69)
Age (y)	Mean (SD)	29.2 (8.1)	28.5 (8.9)
	Range	17–48	16–50
Body mass, kg		81.0 (12.0)	78.8 (13.1)
Body mass index (kg/m^2^)	Mean (SD)	25.8 (2.9)	25.2 (3.2)
	Range	17.9–37.8	19.7–33.7
Time injury to surgery (weeks)	Mean (SD)	14.2 (20.9)	12.1 (15.1)
	Range	2–150	2–104
Sex (males)	*n* (%)	41 (66.1%)	40 (58.0%)
Injured knee is dominant limb	*n* (%)	27 (43.5%)	30 (43.4%)
Injured activity			
Pivoting sports	*n* (%)	59 (89%)	63 (91%)
Other	*n* (%)	7 (11%)	6 (9%)
Injury mechanism (non‐contact)	*n* (%)	61 (92%)	60 (87%)
Graft size (mm)	Mean (SD)	8.5 (0.5)	8.9 (0.5)
	Range	8–10	8–10
Prior contralateral ACLR	*n* (%)	2 (3.2%)	2 (2.9%)
Concomitant surgery			
Meniscus repair	*n* (%)	38 (61.3%)	41 (59.4%)
Lateral tenodesis	*n* (%)	2 (3.2%)	7 (10.1%)
PROMs			
IKDC		51.4 (16.1)	48.9 (16.3)
LKS		61.8 (18.0)	57.5 (17.7)
CKRS		56.2 (18.5)	49.1 (18.6)
VAS‐F		4.0 (2.3)	4.9 (2.5)
VAS‐S		4.3 (1.8)	4.2 (2.0)

Abbreviations: CKRS, Cincinnati Knee Rating System; IKDC, International Knee Documentation Committee Subjective Knee Form; kg, kilograms; kg/m^2^, kilograms per metre squared; LKS, Lysholm Knee Score; mm, millimetres; n, number; PROM, patient‐reported outcome measure; SD, standard deviation; VAS‐F, visual analogue score for pain frequency; VAS‐S, visual analogue score for pain severity; y, years; %, percentage.

### Donor‐site morbidity, PROMs and satisfaction

At 6 months post‐surgery, a significantly lower patient‐reported hamstring pain (*p* < 0.001) and DFPACLR (*p* < 0.001) score was reported for the ST versus STG group (Table 2). A significant group × time interaction was observed for VAS severity (*F*(1, 106.95) = 8.218, *p* = 0.005), with no main effect of group (*F*(1, 107.63) = 0.010, *p* = 0.922) or time (*F*(1, 106.78) = 3.314, *p* = 0.072), adjusting for baseline VAS severity, LET, meniscal repair, sex and age. No differences (*p* > 0.05) were observed between the two groups in other PROMs (Tables 2 and 3). Satisfaction outcomes are shown in Table 4, with no statistically significant group differences observed for any satisfaction item.

**Table 2:** Comparison of 6‐month graft and donor site specific outcomes between the semitendinosus (ST) and semitendinosus/gracilis (STG) autograft groups.

	Group		SMD	*p*
	ST (*n* = 62)	STG (*n* = 68)		
Laxity (SSD, mm)	1.3 (0.9)	1.5 (1.1)	0.14	0.586
DFPACLR (graft morbidity)	16.4 (12.1)	26.1 (15.0)	0.71	<0.001
Hamstring pain	1.1 (1.1)	2.1 (1.3)	0.79	<0.001
ACL‐RSI	43.6 (20.4)	41.6 (19.9)	0.10	0.434

Abbreviations: ACL‐RSI, anterior cruciate ligament return to sport after injury score; DFPACLR, Donor‐site‐related Functional Problems following Anterior Cruciate Ligament Reconstruction; mm, millimetres; SSD, side‐to‐side difference.

**Table 3:** Patient‐reported outcome measures and surgical group comparisons for estimated marginal means and between‐group differences for the intention‐to‐treat analysis, in the semitendinosus (ST) and semitendinosus/gracilis (STG) autograft groups.

Outcome	ST	STG	Mean difference (95% CI)	SMD	*p*
IKDC					
3 months	57.1 (1.6)	55.3 (1.5)	−1.8 (−6.2 to 2.6)	−0.15	0.422
6 months	75.9 (1.3)	72.9 (1.2)	−3.0 (−6.5 to 0.6)	−0.31	0.099
LKS					
3 months	77.1 (1.8)	75.6 (1.7)	−1.4 (−6.3 to 3.5)	−0.08	0.568
6 months	84.2 (1.2)	82.7 (1.1)	−1.4 (−4.6 to 1.7)	−0.12	0.375
CKRS					
3 months	67.5 (1.6)	67.0 (1.5)	−0.5 (−4.0 to 4.9)	−0.03	0.835
6 months	78.5 (1.1)	78.1 (1.1)	−0.3 (−3.5 to 2.8)	−0.03	0.836
VAS‐S					
3 months	2.7 (0.2)	2.3 (0.2)	−0.4 (−1.0 to 0.1)	0.20	0.138
6 months	1.9 (0.2)	2.4 (0.2)	0.5 (−1.0 to 1.0)	0.23	0.084
VAS‐F					
3 months	2.9 (0.3)	3.0 (0.3)	0.1 (−0.7 to 1.0)	0.05	0.723
6 months	2.0 (0.3)	2.3 (0.2)	0.3 (−0.4 to 1.0)	0.10	0.468

Abbreviations: CI, confidence interval; CKRS, Cincinnati Knee Rating System; IKDC, International Knee Documentation Committee Subjective Knee Form; LKS, Lysholm Knee Score; SMD, standardized mean difference; VAS‐F, visual analogue score for pain frequency; VAS‐S, visual analogue score for pain severity; %, percentage.

**Table 4:** Comparison of satisfaction scores between the semitendinosus (ST) and semitendinosus/gracilis (STG) autograft groups.

Outcome	Median (25th to 75th percentiles)		*p*
	ST	STG	
Satisfaction (overall)	1.0	1.0	0.187
Satisfaction (pain relief)	1.0	1.0	0.366
Satisfaction (activities of daily living)	1.0	1.0	0.599
Satisfaction (recreational activities)	1.5	1.0	0.787

### Laxity, strength and hop testing

All patients presented with a normal (or near normal) pivot shift clinical examination at 8 weeks and 4 months post‐surgery, with no Grade II or III pivot laxity outcomes. At 6 months post‐surgery, a significantly higher (*p* < 0.001) LSI was observed for the peak knee flexor strength LSI (Table 5). However, no other group differences were observed in side‐to‐side laxity, or other normalized strength measures or LSIs, for the other strength and hop measures (Table 5).

**Table 5:** Comparison of 6‐month strength and hop outcomes between the semitendinosus (ST) and semitendinosus/gracilis (STG) autograft groups.

	Group		Effect size	*p*
	ST (*n* = 61)	STG (*n* = 68)		
Strength				
Knee extensor strength (Nm/kg)	1.83 (0.51)	1.80 (0.69)	0.05	0.759
Knee extensor symmetry (%)	73.7 (13.1)	71.2 (18.1)	0.16	0.378
Knee flexor strength (Nm/kg)	1.40 (0.37)	1.29 (0.36)	0.30	0.088
Knee flexor symmetry (%)	86.6 (14.1)	77.1 (17.3)	0.59	<0.001
Single hop for distance^a^				
Normalized (m/m)	0.79 (0.20)	0.74 (0.18)	0.23	0.217
Symmetry (%)	0.82 (0.15)	0.81 (0.14)	0.10	0.588

*Note*: Nm/kg = Newton metres per kilogram; m/m = metres hopped as a percentage of metres in height; % = percentage.

a (ST, *n* = 56; STG, *n* = 60).

### Complications and re‐operations

Within the first six post‐operative months, there were no cases of ACL re‐injury. Additionally, there were no documented cases of superficial or deep infections that required hospitalization or treatment with oral or intravenous antibiotics. One patient from the ST group required an arthroscope and manipulation under anaesthesia (MUA) at 3 months post‐surgery. One patient in the STG group also required an arthroscope and MUA at 4 months, while a second STG patient underwent arthroscopy with MUA and debridement of a cyclops lesion at 4 months.

## DISCUSSION

The most important findings of this study are based on the primary end point for which the trial was powered; specifically, that employing an ST versus STG autograft construct resulted in less hamstring pain and hamstring donor site morbidity, and better hamstring strength symmetry. No other differences were observed between the two graft constructs in other PROMs, knee laxity, quadriceps strength or hop capacity.

In support of the first study hypothesis, and to the best of our knowledge for the first time in the available literature, the DOSTAR trial has demonstrated that ST ACLR with suspensory button tibial fixation versus STG ACLR with tibial screw fixation results in a significantly lower level of hamstring pain and donor site morbidity. Numerous studies have highlighted significant morbidity associated with hamstring tendon harvest [6, 7, 8, 22]. However, there is a notable deficiency in comparative data assessing post‐operative donor site morbidity between single and double tendon harvest techniques. In a recent editorial, Rechter et al. emphasized the need for a prospective RCT to assess donor site morbidity and functional outcomes of single versus dual hamstring tendon harvest in ACLR with an 8 mm graft diameter [20]. The DOSTAR trial, which satisfied these criteria, indicates that the STG group experienced significantly higher donor site morbidity compared to the ST group, suggesting that harvesting both tendons leads to increased soft tissue trauma and subsequent morbidity. No other group differences were observed in other subjective outcomes, including PROMs or patient‐reported satisfaction.

The current study demonstrated an improved early recovery of hamstring strength symmetry after ACLR with a ST versus STG autograft construct, supporting the second hypothesis. These results also provide rationale for the potential benefits of preserving the gracilis tendon in maintaining knee flexor function. In contrast, some prior studies have indicated no significant difference between single and double hamstring harvests; however, these studies have primarily been retrospective, often underpowered, and assessed knee flexion predominantly at initial or early ROM angles. A notable case‐control study by Nakamura et al. [18], specifically addressed the deficits in knee flexion comparing single and double hamstring harvests. Their findings revealed that knee flexion was particularly compromised at angles greater than 90°, although the current study focused on peak isokinetic hamstring strength, irrespective of the position in which the peak was attained. They also suggested that knee flexion deficits with dual hamstring tendon harvest are potentially underreported. Other studies have reported knee flexion deficits after ACLR with a hamstring autograft [1, 6, 12, 16, 22]. Despite these findings, most existing studies remain retrospective and lack adequate statistical power. This study represents the first prospective double‐blinded RCT dedicated to investigating these deficits, both from a donor site morbidity and knee flexion strength deficit perspective.

The third and final hypothesis was also supported, with no other group differences observed in other more commonly employed PROMs, rotational (as per the 8‐week and 4‐month pivot shift examinations) or anteroposterior (KT‐1000 scores at 6 months) laxity, quadriceps strength and/or hop testing outcomes. Despite the differences observed in hamstring‐related pain and graft site morbidity, other PROMs employed likely lack specificity to detect differences between two hamstring autograft ACLR cohorts. Furthermore, the quadriceps strength and hop testing mean LSIs at 6 months in the current study, that were well below 90%, are not uncommon for patients undergoing ACLR with a hamstrings autograft [8, 14]. We would suggest that despite the differences in hamstring morbidity and strength, these lingering issues have little effect on quadriceps strength and performance during a SHD.

Despite the double‐blind nature of the study, along with a 100% patient retention rate over the 6‐month period, some study limitations should be acknowledged. First, we recognise the short‐term follow‐up period of 6 months. Nevertheless, the study was developed and powered to investigate early donor site morbidity and knee flexion deficits between the two autograft techniques at 6 months. Longer‐term outcomes will provide an avenue to report on re‐injury and return to sport rates. Second, at this time the study has provided valuable information on the early clinical outcome of ST versus STG ACLR, though no information on the anatomical ramifications of the differing graft harvest techniques. Future work may seek to undertake serial post‐operative MRI to investigate graft signal and hamstring volume, specifically focusing on hamstring reconstitution. This will further enhance our understanding of the regenerative capability of the hamstring tendons following harvest. Third, we acknowledge that rehabilitation can affect the recovery of strength and functional outcomes through the early post‐operative period. This was a community‐level cohort of patients that were provided guidance on rehabilitation from an array of therapists, with this heterogeneity being similar in both surgical groups. Further to that, the study included patients undergoing meniscal repair and LET concomitant with their ACLR procedure. While this may be seen as a limitation (despite the study analysis demonstrating no group‐based differences in these concomitant surgeries), it was the study's intention to recruit an ACLR cohort that was representative of a common community‐level ACLR cohort presenting in clinical practice. Finally, we recognise that utilizing multiple graft construct options provides the surgeon with some flexibility in achieving an optimal graft size. While the option of dual STG tendon autograft is valuable for meeting specific graft size requirements in some patients, the results of this study advocate for the use of the ST/adjustable button as a preferred choice for hamstring grafts when possible, aiming to minimize patient morbidity and enhance overall recovery after ACLR.

In terms of clinical relevance, employing an ST versus STG autograft construct resulted in less hamstring pain and hamstring donor site morbidity, as well as better hamstring strength symmetry. Newer suspensory fixation techniques reliably allow for sufficient graft diameter using only the ST tendon, and the outcomes of the current study would suggest this provides additional benefit with reduced early donor site and functional morbidity.

## CONCLUSIONS

ACLR using an ST autograft resulted in less donor site pain and morbidity, and improved knee flexor strength symmetry at 6 months, while demonstrating comparable functional outcomes to the STG autograft.

## AUTHOR CONTRIBUTIONS

The following authors have conceived and designed the study (Jay R. Ebert, Peter K. Edwards and Peter S. E. Davies), supervised the conduct of the study (all authors), analyzed the data (Jay R. Ebert and Peter K. Edwards), wrote the initial drafts (Adam M. Lawless, Jay R. Ebert, Peter K. Edwards and Peter S. E. Davies), critically revised the manuscript (all authors) and ensure the accuracy of the data and analysis (all authors). The authors confirm that all authors have seen and agree with the contents of the manuscript and agree that the work has not been submitted or published elsewhere in whole or part.

## CONFLICT OF INTEREST STATEMENT

Peter A. D'Alessandro received funding from Smith & Nephew (Research Grant, Fellow Funding, Travel Support, Education Support).

## ETHICS STATEMENT

Ethics approval was obtained by the University of Western Australia Human Research Ethics Committee (RA/4/20/5941). The study was registered with the Australian New Zealand Clinical Trials Registry prior to recruitment (ACTRN12620000927921). Informed and written consent was obtained from all individual participants included in the study.

## Data Availability

Data has not been made publicly available, though data sets generated during the current study can be made available from the corresponding author on reasonable request.
